# Supplementary material for: Proteomics Computational Analyses Suggest that the Antennavirus Glycoprotein Complex Includes a Class I Viral Fusion Protein (α-Penetrene) with an Internal Zinc-Binding Domain and a Stable Signal Peptide
Source: Viruses. 2019 Aug 14;11(8):750. doi: 10.3390/v11080750 (PMC6722660; doi:10.3390/v11080750)
Supplement: Supplementary file 1 [file viruses-11-00750-s001.pdf]

viruses

Supplementary Files

Proteomics Computational Analyses Suggest that the Antennavirus Glycoprotein Complex Includes a Class I Viral Fusion Protein (α-Penetrene) with an Internal Zinc-Binding Domain and a Stable Signal Peptide

Courtney E. Garry [1] and Robert F. Garry[2,3,*]

[1] School of Nursing, Johns Hopkins University, Baltimore, MD 21205, USA; courtneygarry@gmail.com
[2] Department of Microbiology and Immunology, Tulane University School of Medicine, New Orleans, LA 70112, USA
[3] Zalgen Labs, Germantown, MD 20876, USA
* Correspondence: rfgarry@tulane.edu; Tel.: +1-504-988-2027

Table S1. Genbank accession numbers used for sequence and structural analyses.

**Genus**	**Virus**	**Abbreviation**	**glycoprotein**	**nucleoprotein**	**RdRp**	**Zinc-binding protein**
Antennavirus	Wēnlǐng frogfish arenavirus 1	WlFAV-1	YP_009551553.1	AVM87642.1	YP_009551555.1	Not encoded
	Wēnlǐng frogfish arenavirus 2	WlFAV-2	YP_009551607.1	YP_009551604.1	YP_009551605.1	Not encoded
Hartmannivirus	Haartman Institute snake virus 1	HISV-1	AKN10683.2	AKN10684.1	AKN10711.2	Not encoded
	Haartman Institute snake virus 2	HISV-2	AZI72593.1	AZI72594.1	AZI72627.1	Not encoded
	Veterinary Pathology Zurich virus 2	VPZV-2	AZI72595.1	AZI72596.1	AZI72628.1	Not encoded
	Old schoolhouse virus 1	OScV-1	AZI72579.1	AZI72580.1	AZI72620.1	Not encoded
Reptarenavirus	CAS virus	CASV	YP_006590086.1	YP_006590087.1	YP_006590093.1	YP_006590092.1
	University of Giessen virus	UGV	AZI72713.1	AZI72714.1	YP_009508466.1	AZI72641.1
	Golden Gate virus	GGV	YP_006590090.1	YP_006590091.1	YP_006590089.1	YP_006590088.1
	Rotterdam virus	ROUTV	YP_009019194.1	YP_009019195.1	YP_009019197.1	YP_009019196.1
Mammarenavirus (Old World)	Lassa virus	LASV	NP_694870.1	NP_694869.1	AEY85215.1	AEY85214.1
	Lujo virus	LUJV	YP_002929490.1	YP_002929491.1	AFP21519.1	AFP21518.1
	Ippy virus	IPPYV	YP_516230.1	YP_516231.1	YP_516233.1	NC_007906.1
	Wenzhou virus	WENV	YP_009113206.1	YP_009113207.1	YP_009113209.1	AUF72662.1
Mammarenavirus (New World)	Junin Virus	JUNV	ACS12870.1	ACS12871.1	AEB32439.1	AEB32438.1
	Machupo virus	MACV	YP_089665.1	YP_089666.1	YP_089660.1	AIG51561.1
	Guanarito virus	GOTV	ALE15097.1	AAT88083.1	ALE15099.1	NP_899220.1
	Oliveros virus	OLVV	YP_001649210.1	YP_001649211.1	YP_001649214.1	YP_001649215.1

	California (Pichinde) virus	CALV	AAB58485.2	AAD31540.2	AER45496.1	AER45495.1
	Flexal virus	FLEV	YP_001936019.1	YP_001936020.1	YP_001936024.1	YP_001936023.1
	Whitewater Arroyo virus	WWAV	YP_001911113.1	YP_001911114.1	YP_001911120.1	YP_001911119.1
Ebolavirus	Ebola virus	EBOV	AAD14585.1	ALT19749.1	AAD14589.1	Not encoded
	Sudan virus	SUDV	ACR33190.1	AWK96640.1	ALT19784.1	Not encoded
	Bundibugyo virus	BDBV	AKB09565.1	AKB09560.1	ACI28627.1	Not encoded
	Bombali virus	BOMV	ASJ82208.1	QAT98503.1	QAT98511.1	Not encoded
Marburgvirus	Marburg virus	MARV	CAA78117.1	CAA78114.1	ABA87130.1	Not encoded
	Ravn virus	RAVV	ABS17551.1	ACD13016.1	ACD13022.1	Not encoded

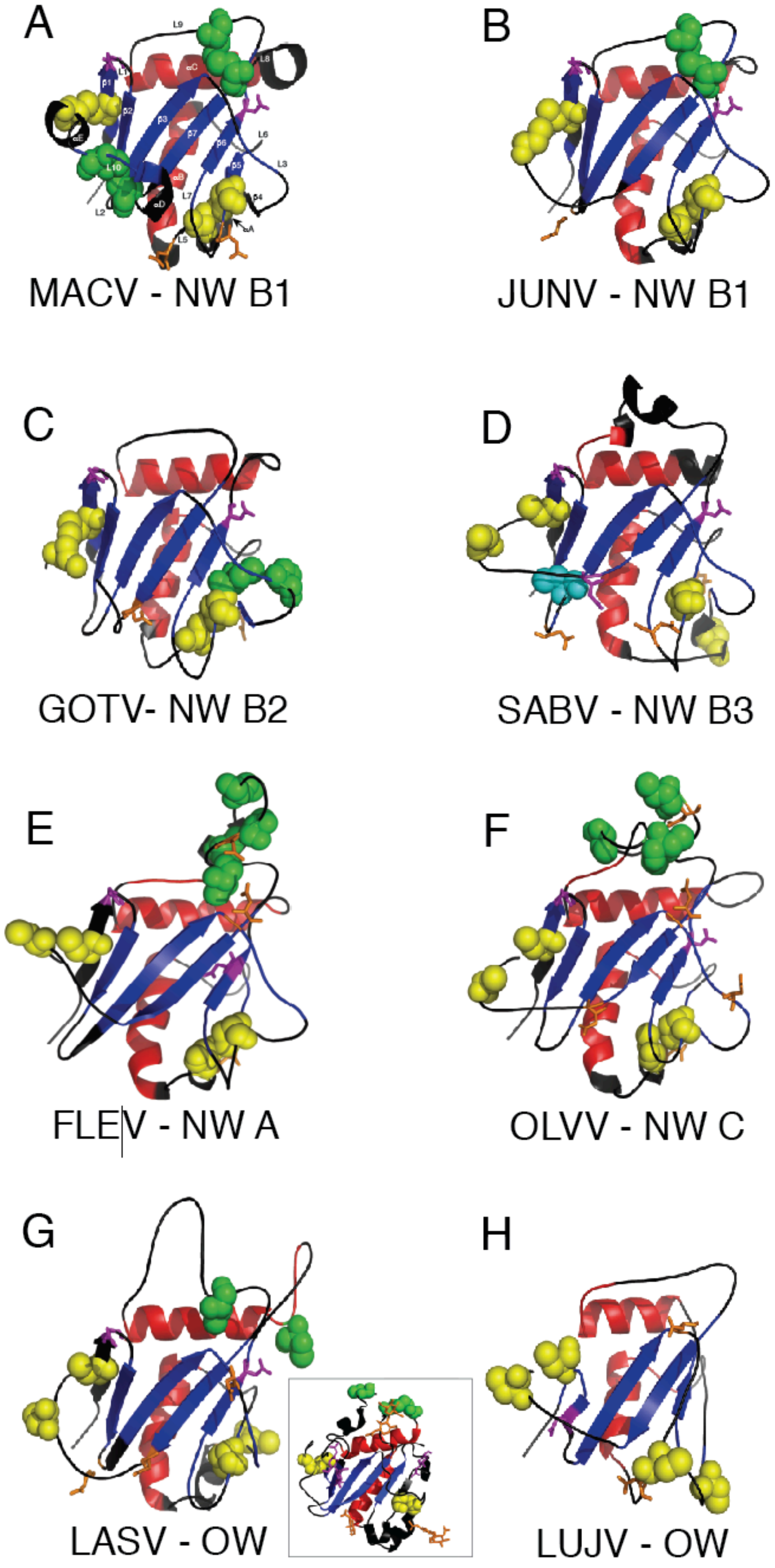

Figure S1. Conserved structure of mammarenaviruses glycoprotein 1. Shown are commonly placed α-helices (red) and a β-sandwich (blue arrows) that comprises the receptor-binding domain. There are two conserved cysteine linkages (yellow) and two conserved N-linked glycans (purple). Virus abbreviations: Machupo MACV, Junin JUNV, Guanarito GOTV, Sabia SABV, Flexal FLEV, Olivaros OLLV, Lassa LASV, Lujo LUJV. Inset: crystal structure of LASV GP1 in the prefusion trimer (PDB 5VK2). Old World OW, New World NW. A, B, C designate sublineages.

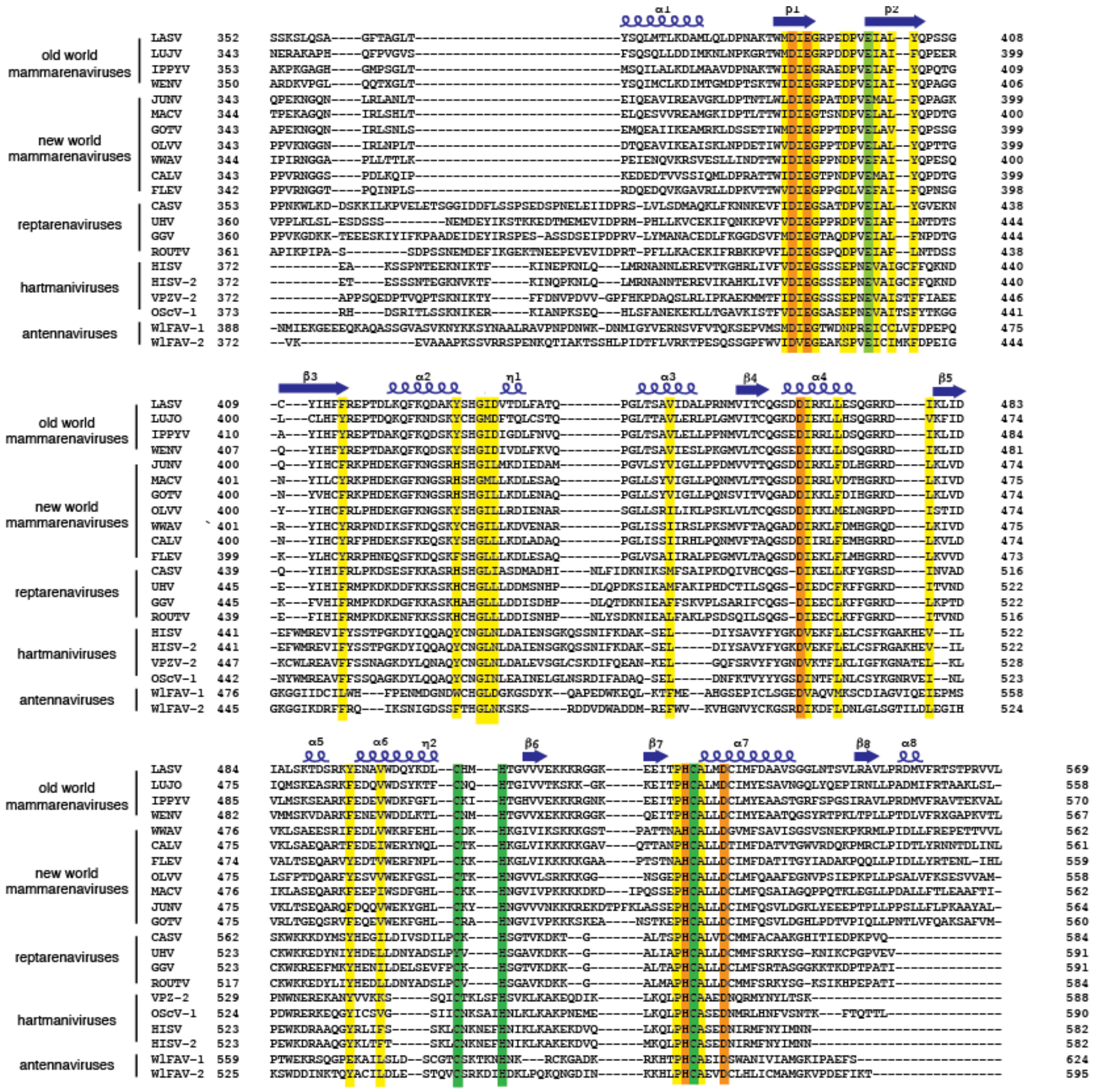

Figure S2. Amino acid sequence alignments of the C-termini of nucleoproteins from representative members of the four arenavirus genera. Residues that are conserved (identical or chemically similar) are highlighted in yellow. Virus abbreviations as in Figure 5. Blue springs represent helical structures and blue arrows are beta sheets. Conserved residues in the active site of the endonuclease (DEDDh box) are highlighted in orange and residues that comprise a conserved a metal binding domain are highlighted in green.

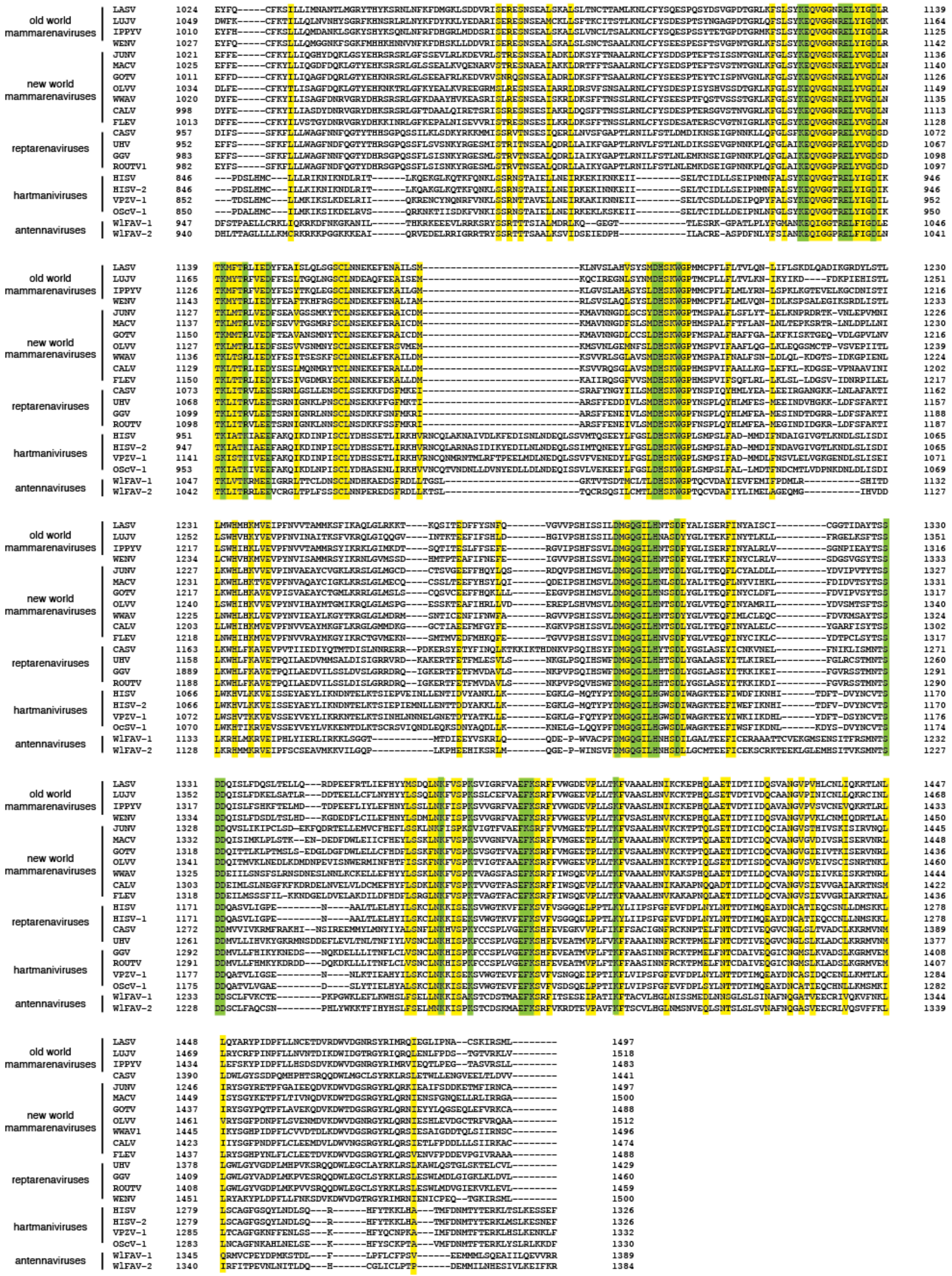

Figure S3. Amino acid sequence alignments of the L protein enzymatic domain from representative members of the four arenavirus genera. Virus abbreviations as in Figure 5. Residues that are conserved (identical or chemically similar) are highlighted in yellow (not required for polymerase activity) or green (required for polymerase activity).

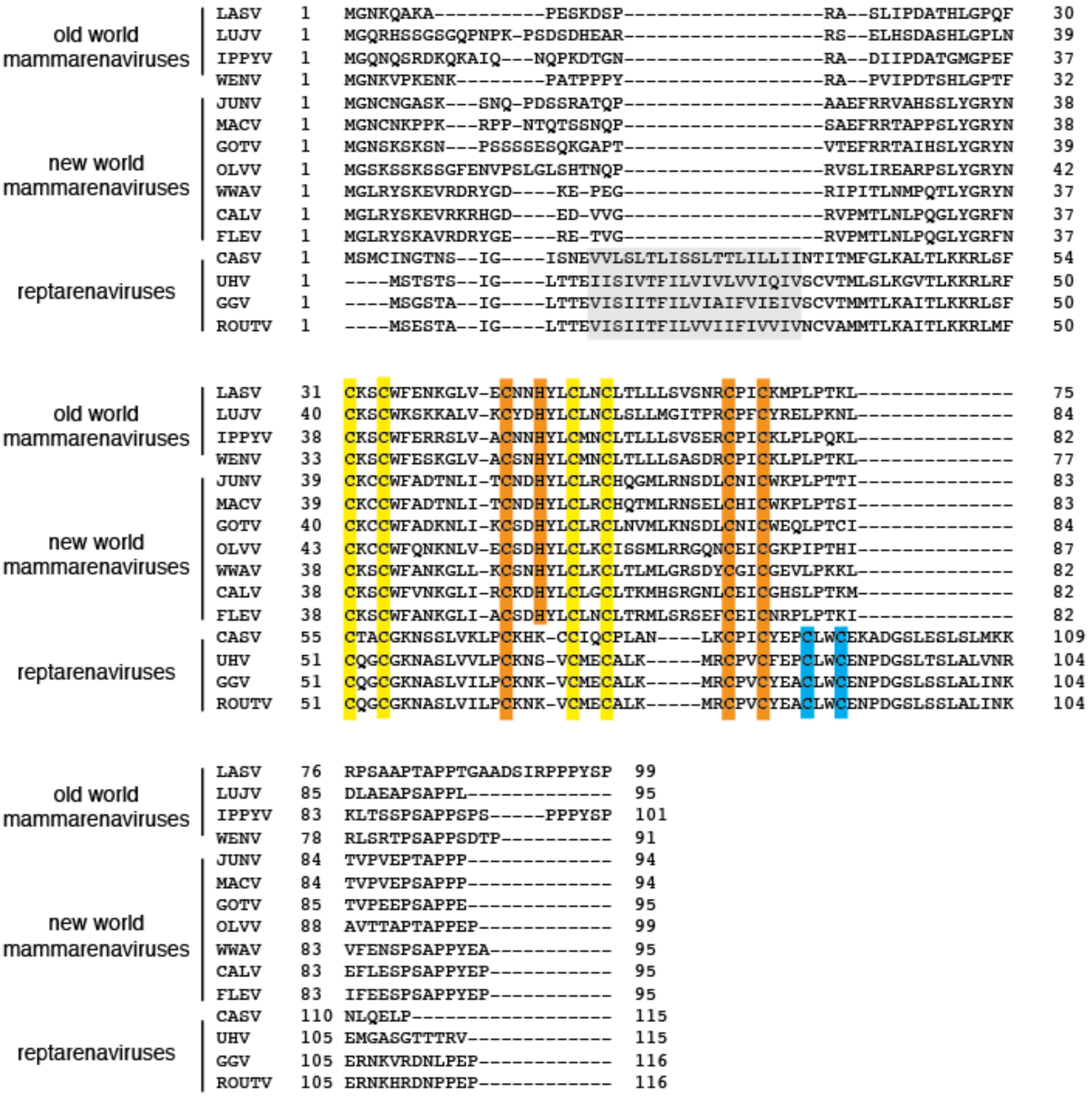

Figure S4. Amino acid sequence alignments of Zinc-binding (matrix) proteins from representative members of the mammarenavirus and hartmanivirus genera. Residues that comprise zinc-binding domain 1 are highlighted in yellow. Residues that comprise zinc-binding domain 2 are highlighted in orange or blue . A transmembrane domain in the Z protein of reptarenavirus is high-lighted in grey. Reptarenaviruses and antennaviruses do not encode a Z protein.
